# Two-year changes in body composition and future cardiovascular events: a longitudinal community-based study

**DOI:** 10.1186/s12986-023-00727-2

**Published:** 2023-01-31

**Authors:** Tingting Hu, Yun Shen, Weijie Cao, Yiting Xu, Yufei Wang, Xiaojing Ma, Yuqian Bao

**Affiliations:** Department of Endocrinology and Metabolism, Shanghai Sixth People’s Hospital Affiliated to Shanghai Jiao Tong University School of Medicine, Shanghai Clinical Center for Diabetes; Shanghai Diabetes Institute, Shanghai Key Laboratory of Diabetes Mellitus, 600 Yishan Road, Shanghai, 200233 China

**Keywords:** cardiovascular event, fat percentage change, fat-free mass percentage change, longitudinal study

## Abstract

**Background:**

The risk of cardiovascular diseases has rapidly increased among middle-aged and elderly. However, little is known about the relationship of body composition changes with the risk of cardiovascular events among this population in China*.* We explored the associations of 2-year changes in fat percentage (fat%) and fat-free mass percentage (FFM%) with subsequent cardiovascular events in a middle-aged and elderly community-based cohort.

**Methods:**

This study included 1048 participants (456 men [43.51%], aged 50–80 years) without overt cardiovascular disease, who underwent two examinations during 2013–2014 and 2015–2016. All participants were followed up until 2022 for cardiovascular events. A bioelectrical impedance analyzer was used to calculate fat% and FFM% change.

**Results:**

At baseline, the median body mass index (BMI), fat%, and FFM% were 23.9 (22.1–25.9) kg/m^2^, 27.2 (20.8–33.6)%, and 72.8 (66.4–79.2)%, respectively. Two-year changes in fat% and FFM% were 0.31 (− 5.53 to 6.87)% and − 0.12 (− 2.36 to 2.06)%. During an average follow-up of 5.5 years, 86 cardiovascular events (8.21%) occurred. Cox regression models showed that hazard ratios (HRs) of every 2% change in fat% and FFM% for cardiovascular events were 1.04 (95% confidence interval [CI] 1.01–1.07) and 0.84 (95% CI 0.74–0.95), respectively. Compared with participants with stable fat% (–2% ≤ ⊿fat% < 2%), those with fat% gain ≥ 2% had an increased risk of cardiovascular events (HR 2.07, 95% CI 1.08–3.97). FFM% loss > 8% was associated with a higher risk of cardiovascular events (HR 3.83, 95% CI 1.29–11.4).

**Conclusions:**

In a middle-aged and elderly community-based Chinese population, fat% gain or FFM% loss was associated with an increased risk of cardiovascular events.

## Background

Obesity is a major global public health concern. According to data from the World Health Organization (2021), the global prevalence of obesity has nearly tripled since 1975 [1]. As the prevalence of obesity continues to rise, the burden of obesity-related comorbidities, especially cardiovascular diseases (CVD), also increases [2, 3]. Body mass index (BMI) is the most widely used index to assess adiposity; however, recent studies have found that individuals with a normal BMI may still be at a high risk for CVD [4]. One possible reason for this may be due to its limited ability to distinguish between fat mass (FM) and fat-free mass (FFM) (mainly skeletal muscle). Thus, the association between body composition and cardiovascular events has attracted considerable attention. Medina-Inojosa et al. reported that among 717 patients with coronary artery disease, the risk of cardiovascular events for those in the highest quartile of fat percentage (fat%) was nearly twice compared with that of the patients in the lowest quartile after a median follow-up of 3.9 years. Conversely, the risk for those in the highest quartile of FFM was 47% lower than that of the patients in the lowest quartile. No association was observed between BMI and cardiovascular events [5]. Another cohort study involving 10,251 diabetic patients demonstrated that after a mean follow-up of 8.8 years, participants in the fourth quartile of the FM index were associated with a 53% increased risk of cardiovascular events compared with those in the first quartile, whereas no significant association was observed between the FFM index and cardiovascular events [6]. Therefore, assessing the role of FM and FFM in the development of CVD may yield new insights into clinical practice. However, most existing studies were somehow limited by single baseline assessment or nonconcurrent measurement at follow-up.

Recent studies have focused on the association between body composition changes and incident CVD. A study involving 5103 patients with type 2 diabetes demonstrated that the decline in FM, but not FFM, was associated with a lower risk of heart failure [7]. However, another type 2 diabetes cohort did not show any significant association of changes in FM and FFM with cardiovascular events [8]. Evidence from participants aged 20–39 years in Korea supported the association of FM and FFM change with cardiovascular events [9]. Nevertheless, there is still a lack of community-based studies in China.

As the risk of high CVD accelerated to increase at the age of 50 years in China [10], studies focusing on middle-aged and elderly population are important. Thus, we aimed to evaluate the relationship of fat% and FFM percentage (FFM%) changes with cardiovascular events in a middle-aged and elderly community-based Chinese cohort.

## Methods

### Study population

**Fig. 1:**
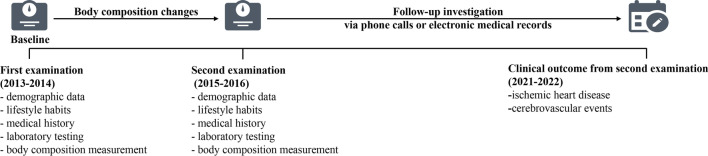
Flow diagram of study population

The participants in this prospective cohort study were recruited from Shanghai communities in 2013–2014. All participants underwent a physical examination, laboratory testing, and body composition measurements at baseline. Information on the history of current and past diseases, medication use, smoking habits, menopausal status, family history, and personal habits was collected using standardized questionnaires at baseline [11]. Those with a validated history of malignant tumors, thyroid dysfunction or hyperthyroidism or hypothyroidism, cardiovascular or cerebrovascular diseases, severe liver or kidney dysfunction, treatment with steroids or thyroxine, age < 50 years, or premenopausal women at enrollment were excluded. In 2015–2016, these participants were invited for a second examination which was similar to the previous. Afterward, participants were followed up during 2021–2022 via telephone or electronic medical records (Fig. 1). From the second examination to the last follow-up, the mean follow-up period was 5.5 ± 0.6 years.

For this study, 1048 participants undergoing two examinations during 2013–2014 and 2015–2016, non-CVD at baseline and during health examination periods, with complete data, were finally included. When compared with non-respondents (including those with missing covariate data or lost to follow-up), respondents had similar BMI, blood pressure, glucose levels, and lifestyle habits. There were fewer men than women in the current study. All participants provided written informed consent. The study was approved by the Ethics Committee of the Shanghai Sixth People’s Hospital affiliated to Shanghai Jiao Tong University School of Medicine.

### Anthropometric and laboratory measurements

Height, weight, waist circumference, and blood pressure were measured using standardized methods. BMI was calculated as the weight in kilograms divided by the squared height in meters. The total FM, fat%, FFM, and FFM% were measured using an automatic bioelectrical impedance analyzer (BIA; TBF-418B; Tanita Corp., Tokyo, Japan). Changes in each body composition compartment (fat% and FFM%) between the first and second examinations were calculated as percentage change [(value2 – value1)/value1 × 100%]. According to previous literature, age-associated muscle loss begins at around 50 years, with mass loss at a rate of 0.5–1.2% per year [12]. Participants were then categorized into four groups:⊿FFM% < – 8%, – 8% ≤ ⊿FFM% < – 2%, – 2% ≤ ⊿FFM% < 2% (reference group: stable), and ⊿FFM% ≥ 2%. In addition, adults who maintain their weight, lose their FFM and gain a similar amount of fat% [13]. Thus, the same classification method was used for fat% change.

Venous blood samples for the measurement of blood routine, glucose, and lipid levels were drawn after a 10-h overnight fast. Participants without a validated history of diabetes underwent a 75-g oral glucose tolerance test, whereas those with diabetes performed a standard bread meal instead. Homeostasis model assessment of insulin resistance (HOMA-IR) was evaluated using the following formula: HOMA-IR = fasting insulin (FINS) (mU/L) × fasting plasma glucose (FPG) (mmol/L)/22.5 [14].

### Outcome

The primary endpoint was a composite of cardiovascular events, including nonfatal myocardial infarction, unstable angina pectoris, hospitalization for heart failure, coronary revascularization, ischemic stroke, and cardiovascular death [15]. The first occurrence of cardiovascular event was recorded via phone calls or electronic medical records in 2021–2022. Subsequently, two well-trained physicians evaluated the outcome data and used the International Classification of Diseases-Tenth Revision (ICD-10) to identify cardiovascular events (I00–I99).

### Diagnostic criteria

Current smokers were defined as participants who smoked at least one cigarette per day for more than six months at baseline [16]. According to the International Physical Activity Questionnaire 2010, physical activity levels were classified as light, moderate, and high [17]. Participants were categorized into two groups: BMI < 25 kg/m^2^ and BMI ≥ 25 kg/m^2^, based on the World Health Organization 2000 criteria [18]. On the basis of the 2020 guidelines of the International Society of Hypertension [19], hypertension was defined as systolic blood pressure (SBP) ≥ 140 mmHg, diastolic blood pressure (DBP) ≥ 90 mmHg, and/or the use of antihypertensive drugs. Diabetes was diagnosed according to the following criteria: (1) FPG ≥ 7 mmol/L, (2) 2 h-plasma glucose (2hPG) ≥ 11.1 mmol/L, (3) glycated hemoglobin A1c (HbA1c) ≥ 6.5%, or (4) a previous diagnosis of diabetes, according to the guidelines of the 2021 American Diabetes Association [20]. Participants with total cholesterol (TC) ≥ 5.2 mmol/L (200 mg/dL), triglyceride (TG) ≥ 1.7 mmol/L (150 mg/dL), low-density lipoprotein cholesterol (LDL-C) ≥ 3.4 mmol/L (130 mg/dL), high-density lipoprotein cholesterol (HDL-C) < 1.0 mmol/L (40 mg/dL), or using lipid-lowering drugs were identified as having dyslipidemia [21].

### Statistical analysis

For continuous variables, normally distributed variables were presented as means ± standard deviations, while skewed variables were described as median and interquartile range. Frequency (proportion) was used for categorical variables. One-way ANOVA, Kruskal–Wallis H test, and chi-square test were used to compare baseline characteristics between fat% change categories for normally distributed, skewed, and categorical variables, respectively. Cox proportional hazards regression analysis was performed to obtain hazard ratios (HRs) and 95% confidence intervals (CIs) of cardiovascular events based on fat% and FFM% changes. Three models were applied: model 1 was adjusted for age and sex; model 2 was further adjusted for lifestyle factors (baseline smoking status, education attainment, family history of CVD, and physical activity); model 3 was further adjusted for metabolic factors (hypertension, diabetes, dyslipidemia, C-reactive protein (CRP), fat%, FFM%, and BMI). The restricted cubic splines with four knots (5th, 35th, 65th, 95th knots) were used to graphically assess the dose–response association between body composition changes and cardiovascular events, because it could better balance both the smoothness of the curve and the accuracy of the model. Interactions of body composition changes with age group (< 65 vs. ≥ 65 years), sex (men vs. women), and overweight/obesity status (BMI < 25 vs. ≥ 25 kg/m^2^) were examined in the fully adjusted model, and HRs with 95% CIs were calculated for each subgroup. All analyses were conducted using R version 4.0.3 (R Foundation for Statistical Computing, Vienna, Austria), and a two-tailed *P* < 0.05 was considered statistically significant.

## Results

### Baseline characteristics of the study population

A total of 1048 participants aged 50–80 years (median, 60; range, 56–63) were analyzed. Of these, 456 were men and 592 were women. The median baseline BMI, fat%, and FFM% for the entire study population were 23.9 (22.1–25.9) kg/m^2^, 27.2 (20.8–33.6)%, and 72.8 (66.4–79.2)%, respectively. The baseline characteristics of the study participants grouped by fat% change categories during the first examination in 2013–2014 are detailed in Table 1. Compared with those with stable fat%, individuals with fat% gain were more likely to be men and current smokers, but with a lower BMI and a lower incidence of hypertension at baseline. Moreover, individuals with fat% gain showed worse metabolic profile changes between first and second examinations (elevated blood pressure, glucose, CRP levels, and poor lipid profiles).

**Table 1:** Clinical characteristics in the first examination in 2013–2014 according to fat% change categories

	Fat% change categories				*P* value
	< – 8%	– 8% to < –2%	– 2% to < 2%	≥ 2%	
*Demographics*					
Men/women, n	101/95	105/116	56/133	194/248	< 0.001
Age, years	60 (55, 63)	60 (56, 64)	61 (57, 64)	60 (56, 63)	0.339
BMI, kg/m^2^	23.2 (21.5, 24.7)	24.4 (22.6, 26.6)	24.2 (22.7, 26.1)	23.2 (21.1, 25.0)	< 0.001
*Social history*					
Above high school, n (%)	119 (60.7)	138 (62.4)	113 (59.8)	273 (61.8)	0.946
Current smoker, n (%)	50 (25.5)	43 (19.5)	25 (13.2)	113 (25.6)	0.003
Family history of CVD, n (%)	62 (31.6)	78 (35.3)	72 (38.1)	150 (33.9)	0.590
Physical activity, n (%)					0.661
Low	38 (19.4)	43 (19.5)	29 (15.3)	84 (19.0)	
Moderate	85 (43.4)	104 (47.1)	87 (46.0)	215 (48.6)	
High	73 (37.2)	74 (33.5)	73 (38.6)	143 (32.4)	
*Comorbidities*					
Hypertension, n (%)	104 (53.1)	124 (56.1)	104 (55.0)	188 (42.5)	0.001
Diabetes, n (%)	38 (19.4)	47 (21.3)	42 (22.2)	85 (19.2)	0.808
Dyslipidemia, n (%)	132 (67.3)	168 (76.0)	149 (78.8)	301 (68.1)	0.010
*Medication*					
Antihypertensive drug, n (%)	49 (25.0)	64 (29.0)	65 (34.4)	117 (26.5)	0.152
Antidiabetic drug, n (%)	19 (9.7)	17 (7.7)	16 (8.5)	37 (8.4)	0.907
Lipid drug, n (%)	20 (10.2)	34 (15.4)	28 (14.8%)	54 (12.2)	0.353
*Metabolic profile changes between first and second examinations*					
SBP, mmHg	− 3 (− 12, 7)	0 (− 10, 12)	− 1 (− 9, 11)	3 (− 7, 12)	0.001
DBP, mmHg	− 3 (− 9, 2)	− 2 (− 8, 4)	− 2 (− 8, 4)	− 1 (− 6, 5)	0.017
FPG, mmol/L	0.42 (0.09, 0.90)	0.41 (0.11, 0.90)	0.66 (0.26, 1.08)	0.75 (0.37, 1.16)	< 0.001
2hPG, mmol/L	− 0.06 (− 1.30, 1.09)	0.30 (− 0.97, 1.66)	0.43 (− 0.89, 1.70)	0.53 (− 0.79, 1.80)	0.009
HbA1c, %	0.10 (− 0.20, 0.30)	0.10 (0.00, 0.30)	0.10 (− 0.10, 0.40)	0.10 (0.00, 0.30)	0.031
FINS, mU/L	− 0.31 (− 2.50, 1.81)	0.47 (− 1.58, 2.45)	0.82 (− 1.31, 3.99)	2.00 (0.08, 4.50)	< 0.001
HOMA-IR	0.04 (− 0.54, 0.70)	0.30 (− 0.29, 0.86)	0.45 (− 0.08, 1.40)	0.77 (0.14, 1.55)	< 0.001
TC (mmol/L)	0.17 (− 0.31, 0.62)	0.17 (− 0.16, 0.59)	0.22 (− 0.18, 0.63)	0.36 (− 0.08, 0.86)	0.001
TG (mmol/L)	− 0.01 (− 0.34, 0.34)	0.09 (− 0.28, 0.55)	0.09 (− 0.30, 0.46)	0.14 (− 0.18, 0.47)	0.011
HDL-C (mmol/L)	0.11 (− 0.02, 0.25)	0.06 (− 0.06, 0.18)	0.05 (− 0.04, 0.15)	0.04 (− 0.09, 0.17)	< 0.001
LDL-C (mmol/L)	− 0.01 (− 0.35, 0.40)	0.10 (− 0.22, 0.41)	0.10 (− 0.21, 0.39)	0.18 (− 0.16, 0.57)	0.008
CRP, mg/L	− 0.01 (− 0.51, 0.43)	0.02 (− 0.37, 0.54)	0.03 (− 0.38, 0.56)	0.12 (− 0.20, 0.71)	0.003

*Fat%* fat percentage, *BMI* body mass index, *CVD* cardiovascular disease, *SBP* systolic blood pressure, *DBP* diastolic blood pressure, *FPG* fasting plasma glucose, *2hPG* 2 h plasma glucose, *HbA1c* glycated hemoglobin A1c, *FINS* fasting insulin, *HOMA-IR* homeostasis model assessment insulin resistance index, *TC* total cholesterol, *TG* triglyceride, *HDL-C* high-density lipoprotein cholesterol, *LDL-C* low-density lipoprotein cholesterol, *CRP* C-reactive protein

### Fat% or FFM% change and cardiovascular events

**Fig. 2:**
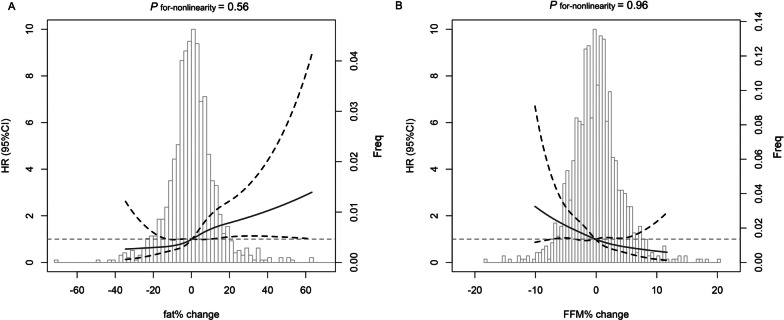
Restricted cubic splines curves for the association of changes in fat% (**A**) and FFM% (**B**) with incident cardiovascular disease. Solid lines represent hazard ratio and dotted regions represent 95% confidence interval. Hazard ratios (95% confidence interval) were calculated by multivariate Cox proportional hazards regression models. The models were adjusted for age, sex, baseline smoking status, education, family history of cardiovascular disease, physical activity, hypertension, diabetes, dyslipidemia, C-reactive protein, fat%, FFM%, and body mass index

During a mean follow-up of 5.5 years after two examinations, 86 (8.21%) cardiovascular events occurred. The median fat% change and FFM% change for the total population were 0.31 (− 5.53–6.87)% and − 0.12 (2.36–2.06)%, respectively. The restricted cubic spline model (Fig. 2) showed that an increase in fat% change was positively associated with a higher risk of cardiovascular events, whereas a decrease in FFM% change was associated with an increased risk of cardiovascular events.

After controlling for the potential confounding factors in the Cox regression model (Table 2), a 2-year change in fat% was significantly and positively associated with the risk of cardiovascular events (HR 1.04, 95%CI 1.01–1.07). Participants with fat% gain ≥ 2% had a significantly increased risk (HR 2.07, 95%CI 1.08–3.97) compared with those with stable fat% (− 2 to 2%).

**Table 2:** Multivariate Cox proportional-hazards analysis showing hazard ratios of fat% change and FFM% change with cardiovascular events

	< –8%	– 8 to < – 2%	– 2 to < 2%	≥ 2%
⊿fat% categories*				
Cases/participants, n	13/196	16/221	12/189	45/442
Model 1	1.07 (0.48, 2.37)	1.01 (0.48, 2.14)	Reference	1.92 (1.01, 3.66)
Model 2	1.08 (0.49, 2.40)	1.02 (0.48, 2.16)	Reference	2.03 (1.06, 3.87)
Model 3	1.13 (0.50, 2.53)	1.01 (0.48, 2.16)	Reference	2.07 (1.08, 3.97)
⊿FFM% categories*				
Cases/participants, n	4/26	20/269	45/480	17/273
Model 1	3.05 (1.07, 8.68)	0.90 (0.53, 1.54)	Reference	0.61 (0.35, 1.06)
Model 2	3.37 (1.18, 9.65)	0.95 (0.56, 1.63)	Reference	0.62 (0.35, 1.08)
Model 3	3.83 (1.29, 11.4)	1.01 (0.58, 1.74)	Reference	0.65 (0.37, 1.14)

Model 1: adjusted for age and sex;

Model 2: further adjusted for current smoking, education attainment, family history of cardiovascular disease, and physical activity;

Model 3: further adjusted for hypertension, diabetes, dyslipidemia, C-reactive protein, basal fat%, basal FFM%, and body mass index

*All covariates in models were from the data collected in 2013–2014

With regard to FFM%, a 2-year change in FFM% was significantly and inversely associated with the risk of cardiovascular events (HR 0.84, 95%CI 0.74–0.95). Participants with FFM% loss > 8% had a higher risk of cardiovascular events than those with stable FFM% (HR 3.83, 95%CI 1.29–11.4).

### Association between fat% and FFM% change and cardiovascular events stratified by different characteristics

**Fig. 3:**
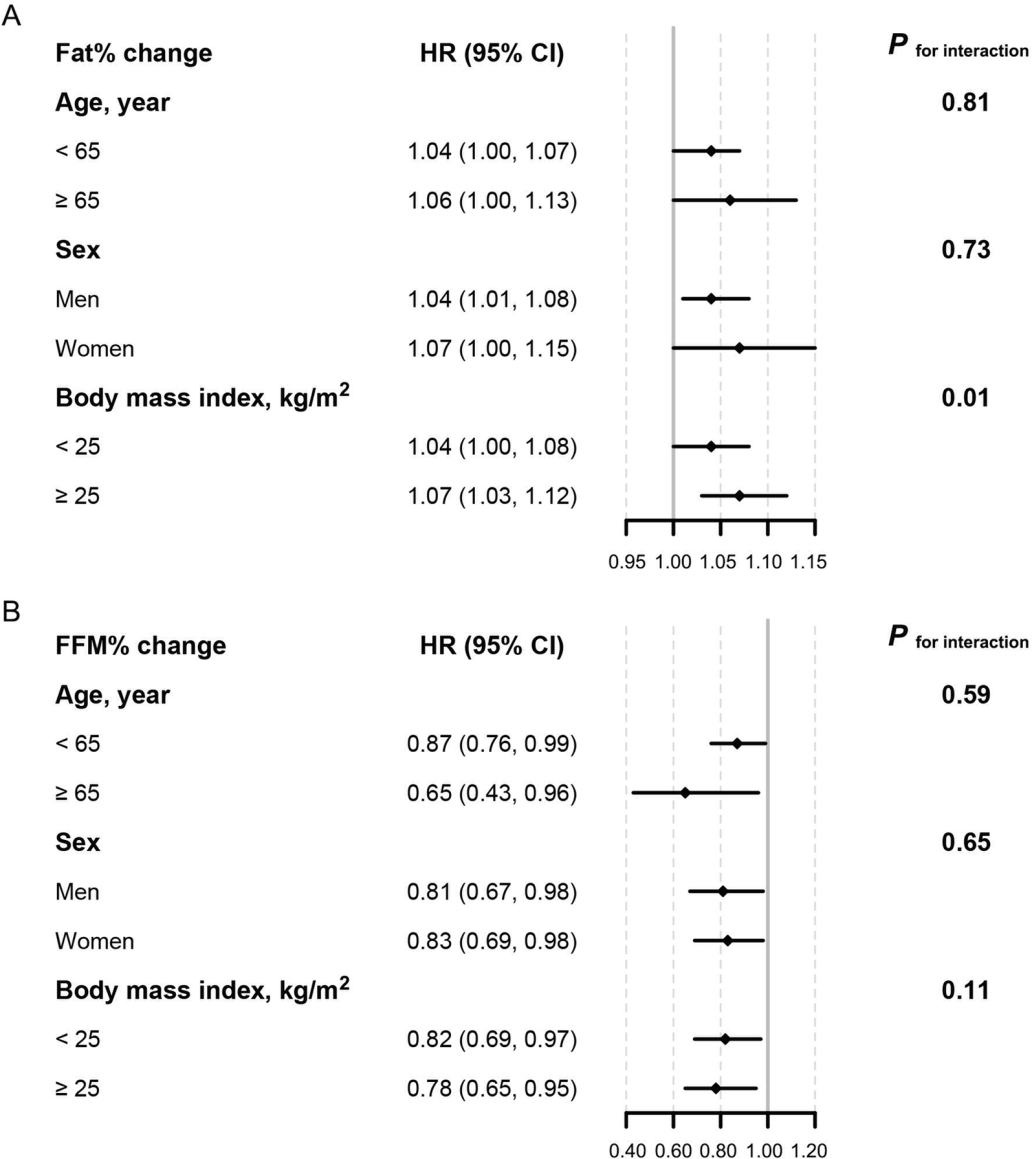
Effects of fat% and FFM% changes on cardiovascular events in different subgroups. Hazard ratios were estimated per 2% increase of fat% and FFM% change. **A** Fat% change and the risk of cardiovascular events. **B** FFM% change and the risk of cardiovascular events. The models were adjusted for age, sex, baseline smoking status, education, family history of cardiovascular disease, physical activity, hypertension, diabetes, dyslipidemia, C-reactive protein fat%, FFM% and body mass index, except the stratified variable

Stratified analyses were performed to evaluate the association of fat% and FFM% changes with cardiovascular events, respectively (Fig. 3). The associations were preserved when participants were categorized by age, sex, and overweight/obesity status. We further observed that BMI modified the association between fat% change and cardiovascular events (*P*_for interaction_ = 0.01). A stronger effect of fat% change on cardiovascular events was observed among participants with BMI ≥ 25 kg/m^2^ (HR 1.07, 95%CI 1.03–1.12) compared with those with BMI < 25 kg/m^2^ (HR 1.04, 95%CI 1.00–1.08).

## Discussion

To the best of our knowledge, this is the first study to explore the association of changes in body composition and cardiovascular events by separating fat% and FFM%, instead of using BMI, which is a composite of muscle and FM, in middle-aged and elderly Chinese populations. In this community-based prospective study, we found that 2-year fat% gain or FFM% loss was associated with an elevated risk of cardiovascular events in the next 5.5 years.

CVDs remain the leading cause of premature mortality in the world, accounting for an average of 17.9 million deaths annually [22]. In China, the burden of CVD has rapidly and substantially increased. According to some estimates, the incidence of cardiovascular events is expected to increase by 50% from 2010 to 2030 [23]. Therefore, tackling CVD risk factors remains a key goal in efforts to prevent, control, and reduce the incidence of CVDs worldwide. Obesity, an estimated risk factor for CVD, has been proven to underlie the development of many metabolic disorders, including hypertension, type 2 diabetes, dyslipidemia [24], and even cardiovascular events [3]. As a common index for measuring adiposity, dynamic changes of BMI have received much attention in predicting cardiovascular events. Choi et al. reported that every 1 kg/m^2^ increase in BMI was associated with a nearly 50% higher risk of acute myocardial infarction or coronary heart disease in the following 9 years [25]. However, BMI alone cannot distinguish body composition. FM plays a detrimental role, whereas FFM is protective [5]. Therefore, it is crucial to accurately evaluate changes in body composition to better understand the association between adiposity and cardiovascular events.

Several prospective studies have explored the association between body composition changes and cardiovascular events. One cohort study recruited over 3 million community-dwelling participants aged 20–39 in Korea [9]. They found that each 1 kg/m^2^ increase in the 2-year FM index change was associated with an elevated risk of cardiovascular events, whereas each 1 kg/m^2^ increase in the FFM index change was associated with a reduced risk of cardiovascular events, which was consistent with our study. Inconsistent with our results, Xing et al. [8] observed that changes in FM and FFM conferred no excess risk of cardiovascular events in 9234 type 2 diabetes patients. Patel et al. [7] found that a 10% increase in FM, not FFM, was associated with a 20% higher risk of heart failure in 5145 participants with type 2 diabetes and overweight/obesity in an American population. The discrepancies may be due to the differences in the included participants and the outcome definition. Participants in the two studies mentioned above were patients with type 2 diabetes, whereas the prevalence of diabetes in our cohort was only 20.2%; this may contribute to different association patterns between body composition changes and cardiovascular events. Therefore, the association between body composition changes and cardiovascular events is still inconclusive. Our cohort study in middle-aged and elderly Chinese residents demonstrated that a 2-year fat% gain or FFM% loss was closely associated with an increased risk of cardiovascular events in a 5.5-year of follow-up. Participants with ≥ 2% fat gain had a 1.07-fold higher risk of cardiovascular events than those with a stable fat%. Also, an FFM% loss > 8% was related to a 2.83-fold higher risk of cardiovascular events compared with the stable group.

The findings of this study have some important implications for the prevention and control of CVD in China. Unlike weight gain, which is usually due to the increase in FM, weight loss may have two causes: FM loss or FFM loss (mainly muscle mass loss). In our analysis, we observed that > 8% of FFM% loss was related to a higher risk of cardiovascular events, and the risk in participants with decreased FM% was similar to that in participants with stable FM%. Our findings imply that one possible reason for the negative effect of weight loss on CVD in some researches [26, 27] might be due to the loss of muscle mass more than the loss of fat mass. More importantly, it has been demonstrated that total muscle mass peaks at the age of 24 years. Afterward, muscle mass is well maintained as about 10% loss occurred between 24 and 50 years. But between 50 and 80 years, an additional loss of 30% occurs [28]. Although aging is inevitable, body composition can be contained. Hence, for middle aged and elderly population, who commonly experience sarcopenia, more attention should be paid to the causes of weight loss. Our findings emphasize the importance of body composition monitoring for better CVD management. Furthermore, these associations held both for middle-aged and elderly population. Thus, it is never too late to initiate control of body composition changes, even in old age.

For middle-aged and elderly population, a reduction in muscle mass is often accompanied by an increase in fat, and vice versa [29]. Crosstalk between body fat and muscle contributes to negative feedback, which in turn results in the development of CVD. The mechanisms of obesity and muscle function in CVD have been well-reviewed. First, excessive fat accumulation promotes the secretion of pro-inflammatory factors, including TNF-α, IL-6, and IL-1, which upregulate IKK/NF-κB and MAPK pathways to induce cell apoptosis and ultimately contribute to myocardial injury [30, 31]. Second, fat and muscle tissues act as endocrine organs that release diverse cytokines, such as adiponectin and Fstl1. Adiponectin, released from adipocytes, facilitates insulin sensitivity by promoting glucose uptake in skeletal muscle and activating the AMPK pathway [32]. However, serum adiponectin levels decrease with increasing body fat. Likewise, Fstl1 functions as a myokine that modulates endothelial function, adverse cardiac remodeling, and subsequent CVD [33]. The activation of oxidative stress [34] and the sympathetic nervous system [35] are also involved in the progression of CVD.

Our study has several limitations. First, it only included the Shanghai population. Further studies are required to verify these findings in different ethnic groups. Second, dietary information was not included in the analyses and will be supplemented in the future. Third, FM and FFM were measured by BIA, not by dual-energy X-ray absorptiometry. However, validation studies revealed that BIA and dual-energy X-ray absorptiometry had high correlations in both men and women (ICC > 0.8) [36]. In addition, BIA has advantages in that it is easy, low-cost, and non-invasive, and is supported by the Asian Working Group of Sarcopenia in community-dwelling settings [37].

## Conclusions

In summary, this longitudinal cohort study revealed that fat% gain or FFM% loss significantly increased the risk of cardiovascular events in middle-aged and elderly, community-based Chinese populations. Thus, changes in body composition should be monitored frequently as an early warning of CVD.

## Data Availability

The datasets generated and/or analyzed during the current study are available from the corresponding author on reasonable request.

## Abbreviations

CVD: Cardiovascular disease
BMI: Body mass index
FM: Fat mass
FFM%: Fat-free mass percentage
Fat%: Fat percentage
BIA: Bioelectrical impedance analyzer
HR: Hazard ratio
HOMA-IR: Homeostasis model assessment of insulin resistance
FINS: Fasting insulin
FPG: Fasting plasma glucose
ICD-10: International Classification of Diseases of Tenth Revision
SBP: Systolic blood pressure
DBP: Diastolic blood pressure
2hPG: 2 h-plasma glucose
HbA1c: Glycated hemoglobin A1c
TC: Total cholesterol
TG: Triglyceride
LDL-C: Low-density lipoprotein cholesterol
HDL-C: High-density lipoprotein cholesterol
CRP: C-reactive protein
CI: Confidence interval
